# PSMC2 is up-regulated in osteosarcoma and regulates osteosarcoma cell proliferation, apoptosis and migration

**DOI:** 10.18632/oncotarget.13511

**Published:** 2016-11-23

**Authors:** Mingzhi Song, Yong Wang, Zhen Zhang, Shouyu Wang

**Affiliations:** ^1^ Department of Orthopaedics, The First Affiliated Hospital of Dalian Medical University, 116011, Dalian, Liaoning, People's Republic of China; ^2^ Department of Orthopaedics, The Third Affiliated Hospital of Dalian Medical University, 116200, Jinpu New Area, Liaoning, People's Republic of China; ^3^ Department of Orthopaedics, Affiliated Central Hospital of Shenyang Medical College, 110024, Shenyang, Liaoning, People's Republic of China

**Keywords:** osteosarcoma, PSMC2, carcinogenesis, oncogenes

## Abstract

Proteasome 26S subunit ATPase 2 (PSMC2) is a recently identified gene potentially associated with certain human carcinogenesis. However, the expressional correlation and functional importance of PSMC2 in osteosarcoma is still unclear. Current study was focused on elucidating the significance of PSMC2 on malignant behaviors in osteosarcoma including proliferation, apoptosis, colony formation, migration as well as invasion. The high protein levels of PSMC2 in osteosarcoma samples were identified by tissue microarrays analysis. Besides, its expression in the levels of mRNA and protein was also detected in four different osteosarcoma cell lines by real-time PCR and western blotting separately. Silencing PSMC2 by RNA interference in osteosarcoma cell lines (SaoS-2 and MG-63) would significantly suppress cell proliferation, enhance apoptosis, accelerate G_2_/M phase and/or S phase arrest, and decrease single cell colony formation. Similarly, pharmaceutical inhibition of proteasome with MG132 would mimic the PSMC2 depletion induced defects in cell cycle arrest, apoptosis and colonies formation. Silencing of PSMC2 was able to inhibit osteosarcoma cell motility, invasion as well as tumorigenicity in nude mice. Moreover, the gene microarray indicated knockdown of PSMC2 notably changed a number of genes, especially some cancer related genes including ITGA6, FN1, CCND1, CCNE2 and TGFβR2, and whose expression changes were further confirmed by western blotting. Our data suggested that PSMC2 may work as an oncogene for osteosarcoma and that inhibition of PSMC2 may be a therapeutic strategy for osteosarcoma treatment.

## INTRODUCTION

Osteosarcoma is regarded as the most common and aggressive form of malignant bone tumor, which is with static 5-year overall survival for children and young adults osteosarcoma patients at 60.0-70.0% in the past three decades [1–2]. Although surgery accompanied with systemic chemotherapy was the gold standard for treating osteosarcoma 2, poor prognosis occurs owing to its resistance to chemotherapy [3]. Therefore, it is imperative to discover a novel target for more effective therapeutic interventions towards osteosarcoma.

Large multimeric proteases in adenosine triphosphate (ATP)-dependent manner play primary catalyzing roles in processing degradation of proteins within eukaryotes and bacterial cells [4]. The 26S proteasome is one of the well-known multimeric proteases and consists of the 20S core catalytic subunit and the 19S regulatory subunit [5]. In eukaryotes, the 26S proteasome is extremely necessary for the quick destruction of key intracellular regulatory proteins, such as transcription factors and cell cycle regulators [4]. Moreover, the 26S proteasome controls cell quality by destroying defective proteins from the cytosol and endoplasmic reticulum [4]. Due to multiple activities and its vital role in apoptosis, 26S proteasome has drawn highly interests for basic studies, especially for prospective prevention and treatment of malignancies [7].

Along with the continuous exploring 26S proteasome, substrates are attracting extensive attention. Proteasome 26S subunit ATPase 2 (PSMC2), located in 7q22.1-q22.3 in the genome, is a pivotal member of the 19S regulatory subunit of the 26S proteasome. The proteasome catalyzes the unfolding and translocation of substrates into the 20S core catalytic particle [8–10]. Additionally, as free 20S particle, but not 19S particle, is available in cells, it indicates that 26S proteasome assembly is limited by the level of 19S regulatory subunit [11]. Therefore, the expression of PSMC2 is potentially necessary for the assembly process of 19S and 26S proteasome [12].

Nijhawan et al. ranked PSMC2 as a top one in CYCLOPS Genes, which represent a special subset of cell essential genes related to cancer cell viability [11]. The observation indicated that partial genomic loss of PSMC2 was sought among more than 3000 tumors, rendering a high dependence of cancer cells on the remaining PSMC2 and further suggested PSMC2 could be regarded as a potential target for treating cancer [11]. Previous studies identified the important role of PSMC2 during certain human cancer [13]. For instance, increased expression of PSMC2 was determined in tumor tissues from the p21-HBx transgenic mice [14]. Furthermore, reduction expression of PSMC2 inhibited ovarian cancer cells proliferation [11]. Although PSMC2 was regarded as a novel synthetic lethal interaction relevant to human cancer 13, the functional validation and mechanism discovery for PSMC2 in malignancies originating from the mesenchymal tissue is completely unclear. Here, we described the effect of PSMC2 in osteosarcoma neoplasm occurrence, growth and metastasis, which have never been reported in osteosarcoma.

## RESULTS

### The high expression of PSMC2 protein in osteosarcoma

**Figure 1:**
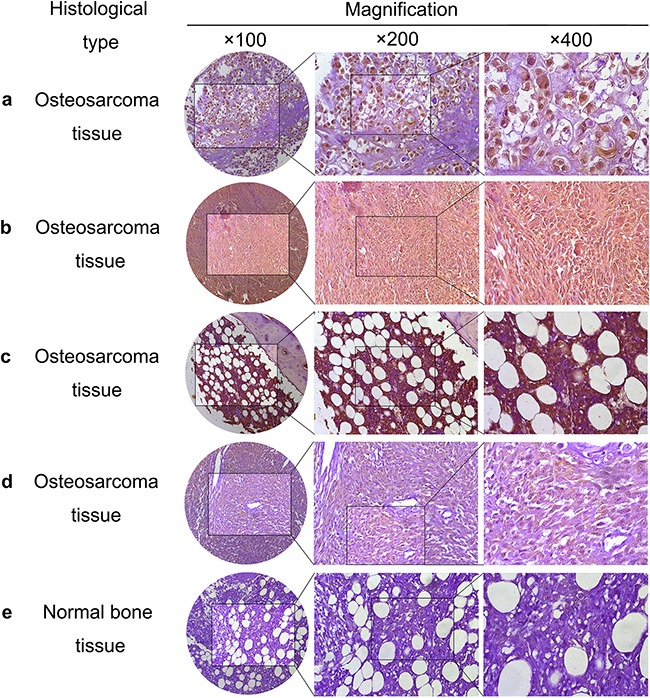
PSMC2 expression in osteosarcoma samples Representative images of osteosarcoma tissue **a-d.** with strong nuclear and cytoplasm staining (brown) and normal bone tissue **e.** with weakly positive PSMC2 staining were shown.

To study the expression of PSMC2 in osteosarcoma, we designed and screened a panel of tissue microarrays including 24 osteosarcoma samples and 5 normal bone samples. 66.7% osteosarcoma samples were with high expression of PSMC2. Compared with osteosarcoma samples, PSMC2 expression was much less in normal bone samples. The detailed information is indicated in Table 1, and Figure 1 shows the representative images of tissue microarrays.

**Table 1:** PSMC2 expression in the patients suffering with osteosarcoma

	Total cases	Cases with high PSMC2 expression	High PSMC2 expression rate (%)
**Age (years)**			
<20	6	2	33.3
≥20	18	14	77.8
**Histology type**			
Chondroblastic	2	2	100.0
Osteoblastoma-like	20	12	60.0
Telangiectatic	1	1	100.0
Fibroblastic	1	1	100.0
**TNM stage**			
T_1_N_0_M_0_	10	6	60.0
T_2_N_0_M_0_	14	10	71.4
**Location of PSMC2 protein expression**			
Nucleus	24	4	16.7
Nucleus and cytoplasm	24	10	41.7
Nucleus, cytoplasm and cytomembrane	24	2	8.3

It is suggested osteosarcoma would be classified into chondroblastic, osteoblastoma-like, telangiectatic and fibroblastic types according to the histological difference. Our tissue microarrays data revealed PSMC2 was highly expressed in all the chondroblastic, osteoblastoma-like, telangiectatic and fibroblastic cases and approximate 60% incidence of strong positive PSMC2 expression was detected. This result may be consent with the consensus that osteoblastoma-like osteosarcoma is more benign than other types. In this study, within T_1_N_0_M_0_ cases, strongly positive PSMC2 cases accounted for 60.0%, whereas this strongly positive rate increased to 71.4% in T_2_N_0_M_0_ osteosarcoma patients. Additionally, PSMC2 was detected to distribute in both nucleus and cytoplasm.

In summary, these results revealed that high levels of PSMC2 expression might be related to osteosarcoma development as well as serve as a marker for osteosarcoma diagnosis.

### Silencing of PSMC2 expression inhibits cell growth *in vitro* as a result of reduced proliferation, enhanced apoptosis and impeded colony formation

**Figure 2:**
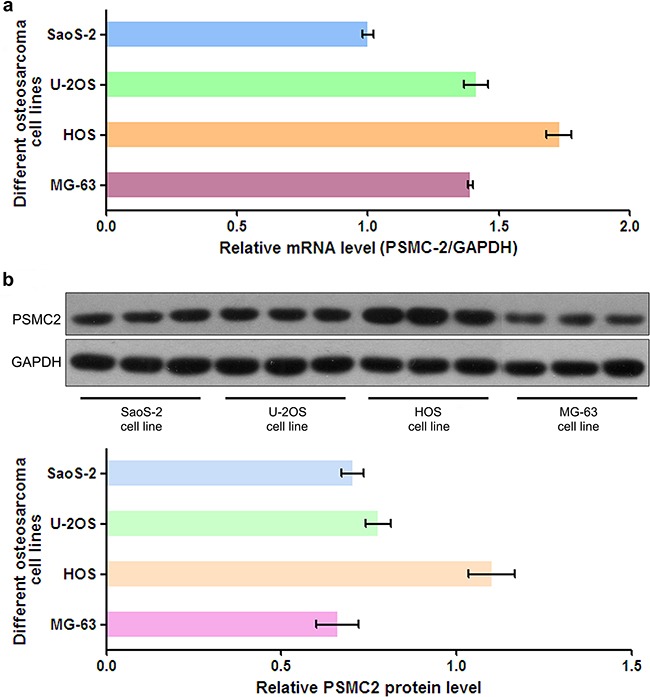
The mRNA level and protein expression of PSMC2 in osteosarcoma cells **a.** PSMC2 mRNA from four common osteosarcoma cell lines was all detected by real-time PCR. **b.** Western blotting showed that PSMC2 expressed in four common osteosarcoma cell lines.

**Figure 3:**
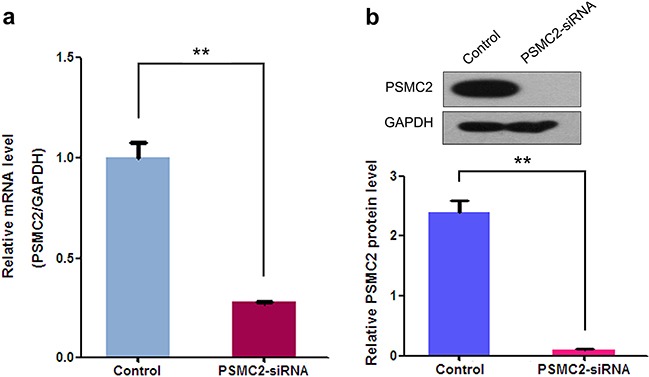
Effects of siRNA mediated PSMC2 knockdown in SaoS-2 osteosarcoma cells Compared to the control, siRNA against PSMC2 was conducted via lentivirus infection and PSMC2 expression in SaoS-2 osteosarcoma cells were determined at both the mRNA levels by real-time PCR and protein level by western blotting. Data were presented as mean ± SD from three independent experiments. **P < 0.01.

To assess PSMC2 expression levels in different osteosarcoma cell lines, mRNA and protein expression of PSMC2 were assessed by a panel of different osteosarcoma cell lines (SaoS-2, U-2OS, HOS and MG-63) via real-time PCR and western blotting (Figure 2). Finally, we selected SaoS-2 and MG-63 cell lines for subsequent studies as their moderate levels of endogenous PSMC2 would be better to represent the expression of PSMC2 in primary human osteosarcoma tissues. Lentivirus-mediated small RNA interference was conducted and suppressed PSMC2 expression levels which were indicated by real-time PCR and western blotting from SaoS-2 cells with five days infection (Figure 3).

**Figure 4:**
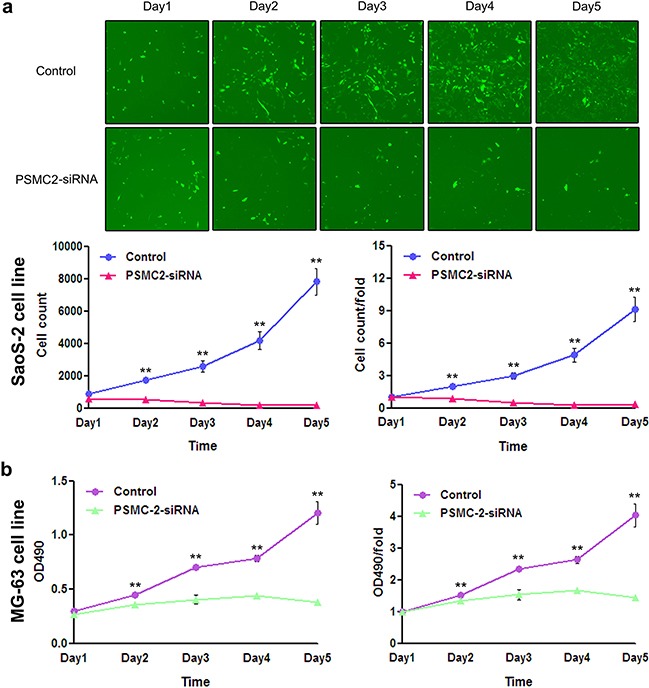
Effect of PSMC2 knockdown on osteosarcoma cell growth **a.** PSMC2 silence in SaoS-2 osteosarcoma cells was established via lentiviral infection. During five days continuous cell counting via fluorescence microscope, the quantity of PSMC2-siRNA SaoS-2 osteosarcoma cells decreased gradually, compared to the control. Histogram represented the number of PSMC2-siRNA SaoS-2 osteosarcoma cells and control cells at indicated times. **b.** MTT assay was used to determine the MG-63 cell growth after PSMC2 knockdown. **P < 0.01 as compared with normal control cells.

**Figure 5:**
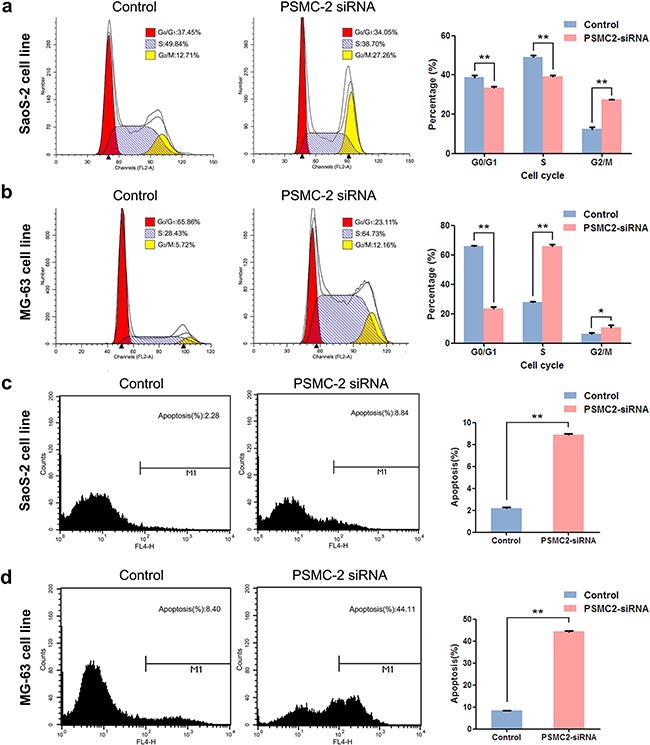
Consequences of PSMC2 silencing on cell cycle progression and apoptosis in osteosarcoma cells **a-b.** Cell cycle was determined in SaoS-2 cells and MG-63 cells by flow cytometry five days after treatment with the indicated si-RNAs. The diagrams quantified cell fractions in the G_0_/G_1_, S and G_2_/M fractions were shown. **c-d.** Apoptosis was determined by flow cytometry assays in two osteosarcoma cell lines with PSMC2 silence and control cells. The apoptotic rate was calculated as the percentage of Annexin FITC positive cells. Data were presented as mean ± SD from three independent experiments. **P < 0.01.

Consequently, knockdown of PSMC2 expression in SaoS-2 osteosarcoma cells and MG-63 osteosarcoma cells was ready to suppress cell growth rate determined by 3-(4,5-dimethyl-2-thiazolyl)-2,5-diphenyl-2-H-tetrazolium bromide (MTT) and fluorescence microscope during five-day cultures (Figure 4). The decreased cell growth could be attributed from impaired cell cycle progression and/or increased cell death. To further verify this issue, we used flow cytometry to analyze cell cycle and apoptosis in PSMC2 silenced osteosarcoma cells. PSMC2 depletion in SaoS-2 cells leads to a reduced cells population in both G1 and S phase as well as a significant arrest in G2/M phase (Figure 5a). Similarly, enhanced G2/M phase arrest was also determined in PSMC2 silenced MG-63 cells but accompanied with an increased cell population in S phase (Figure 5b). Besides, PSMC2 suppression would give rise to a greater acceleration in cellular apoptosis in both SaoS-2 cells and MG -63 cells (Figure 5c and 5d).

**Figure 6:**
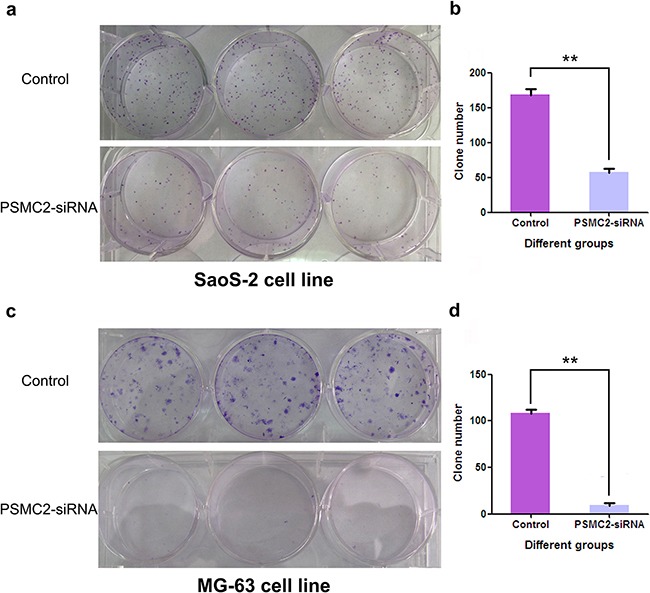
Impact of PSMC2 silencing on osteosarcoma cell colony formation Three days post-transfection, Osteosarcoma cells were embedded in soft agar and grown for 15 days. The resulting colonies (a:SaoS-2 cell line; c:MG-63 cell line) were stained with Giemsa stain. Colonies/well in 6-well plates were counted using software Image J. b.d. Data of PSMC2-siRNA and control cells were the mean ± SD. **P < 0.01. One of three representative experiments was shown.

Colony forming ability is another nature for malignant tumors. Giemsa staining was performed to explore the impacts of PSMC2 silence on colony forming in two different osteosarcoma cell lines with the fifteenth day of cell cultures (Figure 6a and 6c). The cell colony numbers were quantified and demonstrated PSMC2-knockdown was efficiently inhibiting osteosarcoma cell colony formation (Figure 6b and 6d).

### Silencing of PSMC2 inhibits tumorigenicity *in vivo*

**Figure 7:**
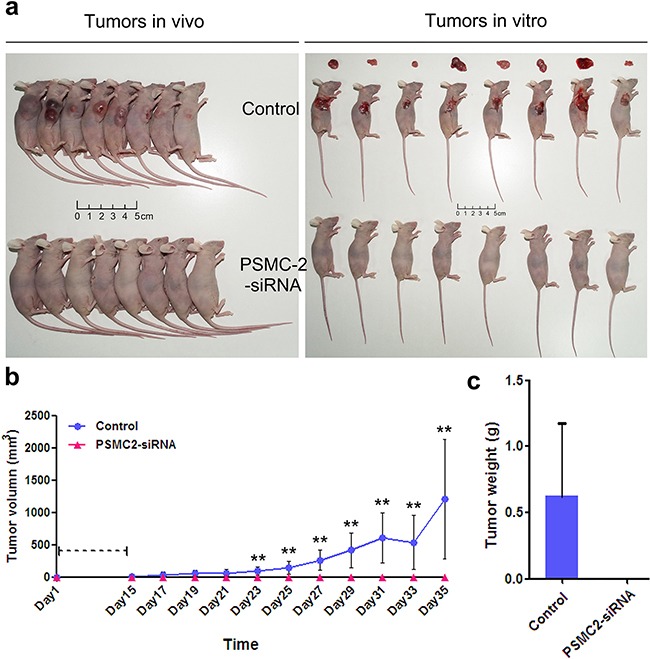
Knockdown of PSMC2 in SaoS-2 cells inhibited tumorigenicity in nude mice An equal number of cells (normal control and PSMC2 knockdown osteosarcoma cells) were inoculated subcutaneously into nude mice for up to 35 days. **a.** After scarification, xenograft tumors in vivo and excised tumors were recorded. **b.** Tumor volume was estimated based on the following equation: volume = 1/2 × (largest diameter) × (smallest diameter)^2^ and data were obtained from eight animals in each group presented as mean value ± SD. **c.** Bar graph presented the excised tumor weights derived from control or PSMC2 silenced SaoS-2 cells. **P < 0.01 as compared with normal control cells.

To further confirm the oncogenic role of PSMC2 *in vivo*, osteosarcoma xenograft mouse models were utilized. Right armpit region of NOD/SCID nude mice was chosen to perform subcutaneous injection with osteosarcoma cells that were infected with either control or PSMC2 siRNA lentiviruses. Then, the development of tumor was observed continuously after cell implantation under overall health status for all individuals (Figures 7b). Tumors were excised and then subjected to measure size and weight. There was no tumor growth in the PSMC2 knockdown group was observed in 35 days (Figures 7a). After scarification, the averaged tumor weight of the control mice was extremely heavier than that in silencing PSMC2 group which presented no tumor growth (Figures 7c). These results clearly indicated that silencing PSMC2 expression inhibited osteosarcoma cell tumorigenicity *in vivo*.

### Down-regulation of PSMC2 decreases the invasion and migration capability of osteosarcoma *in vitro*

**Figure 8:**
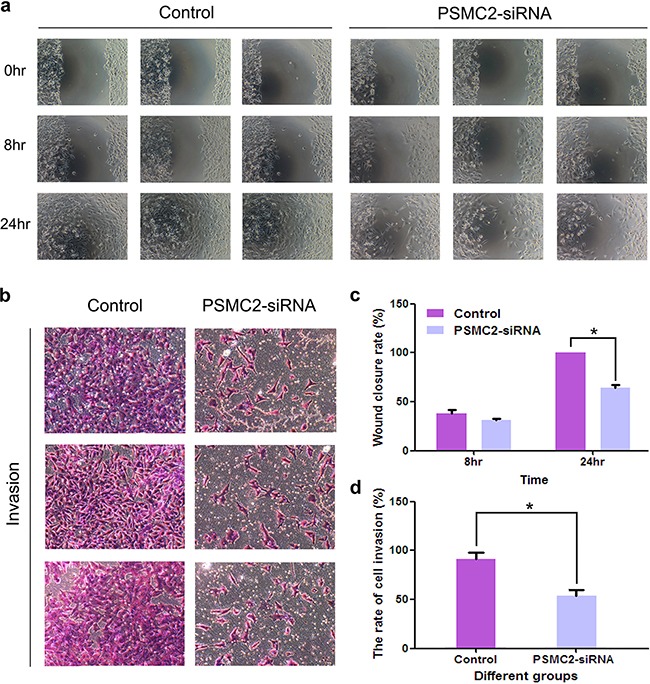
Effects of Silencing PSMC2 on migration and invasion in osteosarcoma cells **a.** The number of migrative cells was determined using scratch would healing assays and the cells migrating progression was recorded under microscope at indicated time points. **c.** Histogram represented the relative wound closure speed at 8 and 24 hours. *P < 0.05 as compared with normal control cells. **b.** Cell invasive ability of control or PSMC2 knockdown cells were determined via a transwell assay. And the representative phase contrast images demonstrated the invasion of the cells. **d.** Bar graph summarized the rate of cell invasion from control and PSMC2 silenced cells *P < 0.05 as compared with normal control cells. All results were confirmed in three independent experiments.

Metastasis is an important characteristic of malignant tumors. A positive correlation between PSMC2 expression and osteosarcoma metastasis was found in this study. Subsequently, we attempted to evaluate whether silencing PSMC2 in osteosarcoma would have an effect on tumor migration and invasion. A much lower wound closure rate was detected via the wound-healing assay in the PSMC2 silenced SaoS-2 cells as compared with control cells (Figure 8a & 8c). Furthermore, significantly reduced invasive ability was also witnessed in SaoS-2 osteosarcoma cells upon PSMC2 knockdown determined by a transwell based invasion assay (Figure 8b & 8d). Our data strongly supported that PSMC2 was greatly required for invasion and migration capability in SaoS-2 osteosarcoma cells.

### Inhibiting proteasome induces osteosarcoma cell growth suppression, increased cell cycle arrest and enhances apoptosis

**Figure 9:**
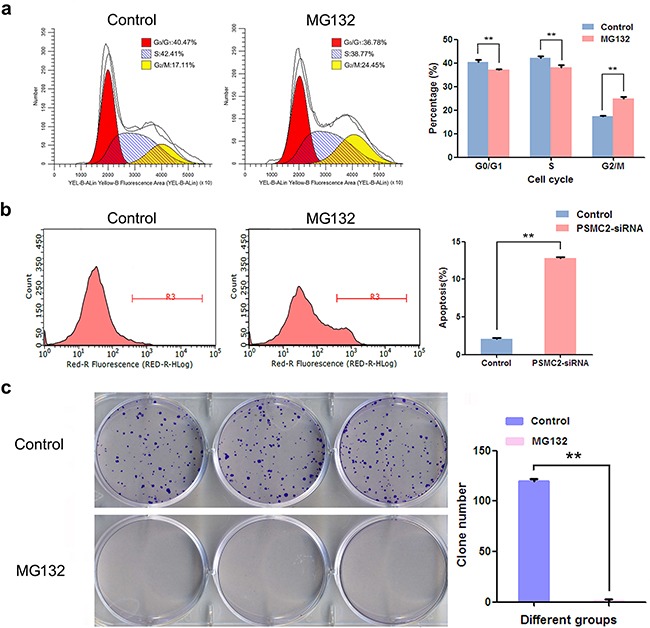
The effect of inhibiting proteasome on cell cycle, apoptosis and colony forming After treatment with MG132, flow cytometry analysis was used to evaluate. **a.** Cell cycle progression and cell fractional quantification in the G_0_/G_1_, S and G_2_/M phases were shown in the diagrams. **b.** Apoptosis via quantifying Annexin FITC positive cells was shown. **c.** Representative pictures showing colonies formation from cells with or without MG132 treatment with Giemsa staining and data were from three independent experiments. **P < 0.01.

As PSMC2 is a crucial element for proteasome assembly and functional performance, we deduced PSMC2 depletion causing biological defects in osteosarcoma cell might associate with disabled proteasome activity. To explore the notion, MG132, a selective inhibitor of the proteasome, was applied to SaoS-2 osteosarcoma cells. Our data revealed MG132 was able to suppress cell population in G_2_/M phase (Figure 9a), promote apoptosis (Figure 9b) as well as extinct single cells derived colonies (Figure 9c). Herein, we proved proteasome is necessary for osteosarcoma growth as its inhibition or deficiency for PSMC2 would lead to similar defects in osteosarcoma biological function.

### Enhanced expression of PSMC2 inhibits osteosarcoma growth

**Figure 10:**
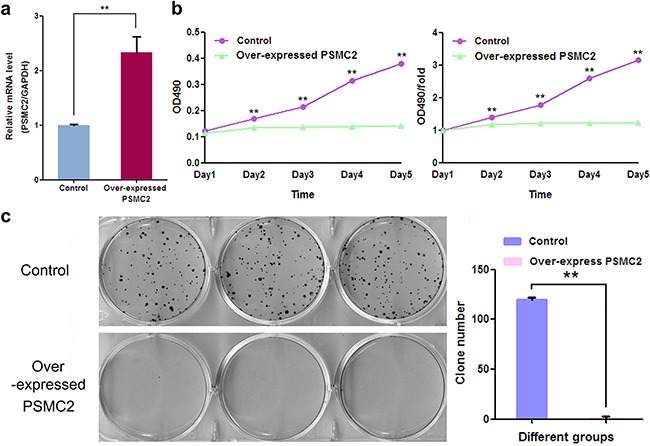
The effect of exogenously enforced PSMC2 expression on osteosarcoma cell growth **a.** The mRNA levels of PSMC2 in control and PSMC2 over-expressing cells were assessed by real-time PCR and the consequent influences on osteosarcoma growth were separately determined by **b.** MTT assay and **c.** colony formation assay. Data were from three independent experiments. **P < 0.01.

To further exam the consequence of enhanced PSMC2 expression on osteosarcoma biological function, lentivirus transduction was used to specifically up-regulate PSMC2 expression in SaoS-2 osteosarcoma cells (Figure 10a). As results, overexpression of PSMC2 exhibited a complete blockage effect on SaoS-2 cell growth during five days assessment via MTT assay. In consistent, colony forming capacities was also totally suppressed in PSMC2 overexpressing cells (Figure 10c).

Overall, the observation above indicated exogenous enhanced PSMC2 expression applied less power on osteosarcoma proliferation and colony forming.

### Silencing of PSMC2 alters oncogene expression in osteosarcoma cells

**Figure 11:**
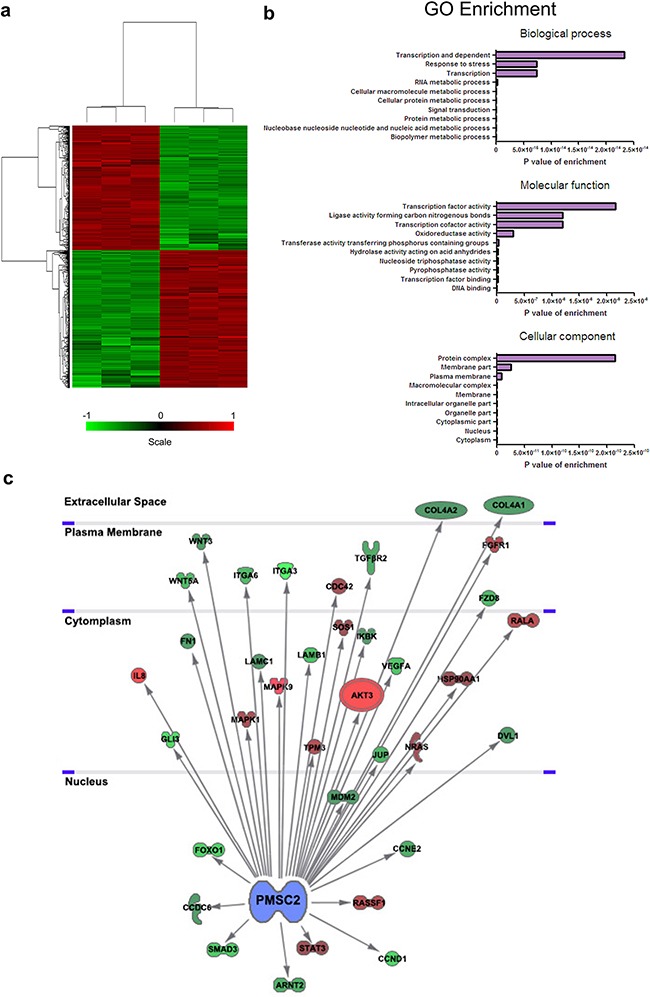
Global changes in osteosarcoma cell transcriptome following knockdown of PSMC2 expression **a.** Hierarchical cluster analysis of PSMC2-siRNA and normal control osteosarcoma cells. A total of 1555 genes were differentially expressed between PSMC2-siRNA and the normal osteosarcoma cells. Heat-map colors represented mean-centered fold change expression in log-space. **b.** Gene ontology analyses of PSMC2-regulated gene expression events. Fisher exact P-values were plotted for each category. **c.** Knowledge-based interaction network of PSMC2 and PSMC2 targets after comparing PSMC2-siRNA cells and control cells. The network was built based on the PSMC2 interactome of microarray data from osteosarcoma cells with a 1.5-fold change cut-off. The intensity of the node color indicates the degree of up- (red) or down- (green) regulation (light color stood for more significant P-values).

To further elucidate the molecular mechanism of PSMC2 in direct osteosarcoma malignance, whole-genome Affymetrix GeneChip hybridization was adopted to discover PSMC2 silencing affected gene expressional pattern and analyze the potential regulation manners. Following bioinformatic and normalization analysis, the two groups (control osteosarcoma cells and PSMC2 knockdown osteosarcoma cells) would be clearly distinguished by hierarchical cluster analysis in Figure 11a as well as principal component analysis. Among 1555 genes whose expression was affected by PSMC2 knockdown, 198 genes reached to the significant difference. We further narrowed down the target genes with greater than one have half times changed, and P-values were less than 0.05, as verified by two-tailed t-test.

Discovery of PSMC2 function and pathway enrichment analyses were conducted by performing Kyoto Encyclopedia of Genes and Genomes (KEGG), Gene Ontology (GO) and BioCarta pathways bioinformatic tools. GO analysis indicated marked alterations between the control and PSMC2 knockdown osteosarcoma. Some significant categories and representative genes expressed differentially and were shown in Figure 11b. Besides changes in expression of proteasome genes set or proteasome pathway related gene set, pathway analysis also emphasized genes involving carcinogenesis pathways (Table 2). Moreover, the interaction network of PSMC2 with those cancer associated genes was constructed (Figure 11c). We determined expression levels of thirty-eight genes related to cancer that might be markedly regulated by PSMC2. Integrin alpha 6 (ITGA6), Fibronectins 1 (FN1), Cyclin D1 (CCND1), Cyclin E2 (CCNE2) and Transforming growth factor-beta type 2 receptor (TGFβR2) were significantly down-regulated by the depletion of PSMC2 (Figure 11c).

**Table 2:** Pathway enrichment gene sets

Pathway enrichment terms	Genes in Gene Set	Genes in Overlap	P-value^b^
KEGG_PROTEASOME	48	20	3.23E-15
BIOCARTA_PROTEASOME_PATHWAY	28	16	4.78E-15
KEGG_PATHWAYS_IN_CANCER	328	38	1.43E-09
KEGG_FOCAL_ADHESION	201	28	7.59E-09
KEGG_ECM_RECEPTOR_INTERACTION	84	18	1.16E-08
KEGG_EPITHELIAL_CELL_SIGNALING_IN_HELIELICOBACTER_PYLORI_INFECTION	68	15	2.14E-07
KEGG_TIGHT_JUNCTION	134	20	6.48E-07
KEGG_NEUROTROPHIN_SIGNALING_PATHWAY	126	18	6.01E-06
KEGG_VIBRIO_CHOLERAE_INFECTION	56	12	7.51E-06
KEGG_P53_SIGNALING_PATHWAY	69	13	1.03E-05

a Based on the gene data of pathways of KEGG and BIOCARTA, all the different genes were managed by enrichment analysis and ranked by the P-value.

b P-value was significance probability.

**Figure 12:**
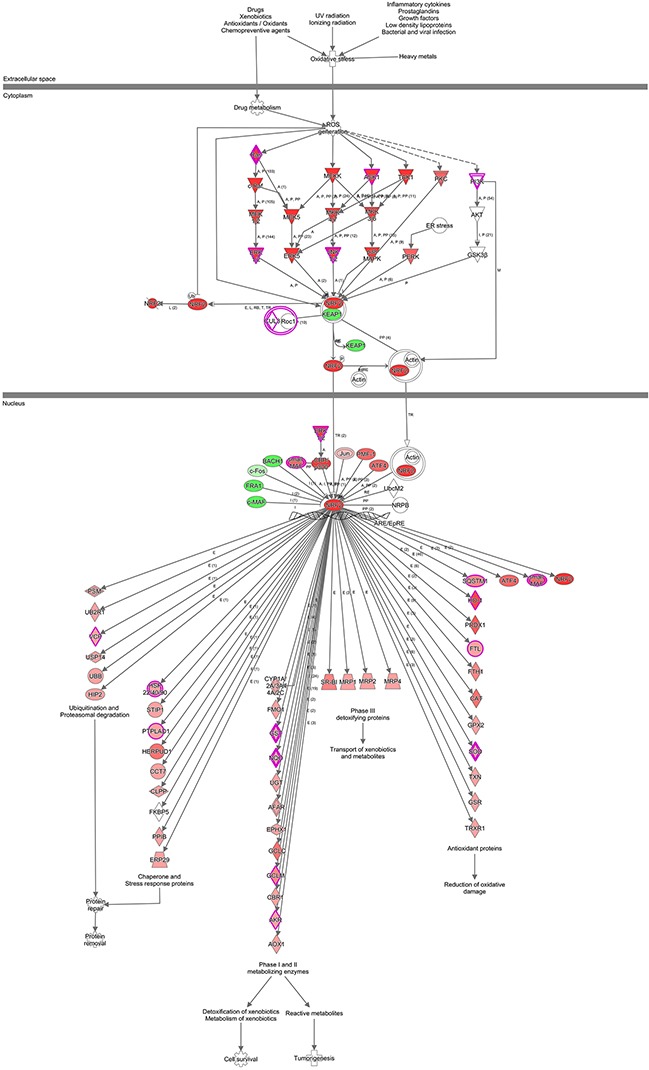
IPA identified proteins networks showing inter-relationships and pathways A sub-network of proteins with different expression and altered pathway was demonstrated. Green fields indicated down-regulated and the red fields indicated up-regulated proteins. For protein network or pathways analysis, statistical significance was determined with Fisher's exact test (P < 0.05).

To forecast the inter-relationships and pathways that are related to PSMC2, we use IPA (Ingenuity pathway analysis) to describe the networks of protein. Although PSMC2 is absent from the networks, it still plays an essential role in the pathways that summarized with this study data and reported by existing literatures (Figure 12).

**Figure 13:**
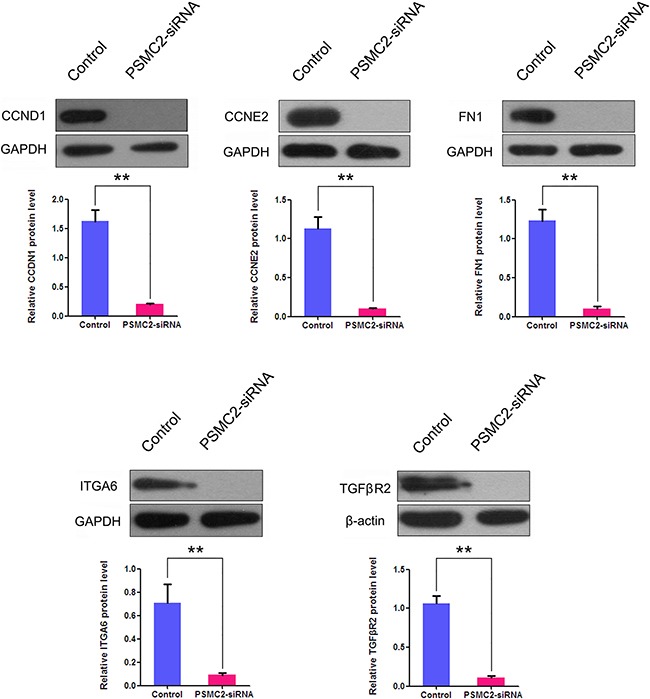
The effects of PSMC2 silence on ITGA6, FN1, CCND1, CCNE2 and TGFβR2 in osteosarcoma cells Western blotting was used to evaluate the protein expressions of ITGA6, FN1, CCND1, CCNE2 and TGFβR2 in PSMC2 silenced osteosarcoma cells and normal osteosarcoma cells. GAPDH and β-actin were used as a loading control. Histogram representing indicated the results of three independent experiments (**P < 0.01).

To further verify the microarray results, we performed western blotting and discovered the proteins expression levels of ITGA, FN1, CCND1, CCNE2 and TGFβR2 were all significantly decreased in PSMC2 knockdown cells in comparison with control cells. (Figure 13).

## DISCUSSION

Osteosarcoma, characterized by a direct formation of bones or osteoid, is the most frequent bone malignancy. It is commonly diagnosed among children and adolescents, although this neoplasm can affect people in any age [15]. According to the current epidemiological analysis, osteosarcoma is prone to grow predominantly on the area of the metaphyseal in young adults or axial locations on elderly patients [16–17]. Whereas, prior studies have demonstrated a poorer prognosis was in both younger patients and aged patients [15]. To our best knowledge, osteosarcoma is a malignant lesion with low responses and resistance to chemotherapy and surgery based complete removal of the lesion represents a better prognosis [18–19]. However, it commonly originates from so-called “hard to access locations” or in areas impossible to access surgically [18]. Consequently, the development of new therapeutic strategies is necessary to achieve a desirable effect on osteosarcoma treatment.

The 26S proteasome has already been treated as a non-lysosomal proteolytic machine of eukaryotes. It consists of a 20S core subunit and a 19S regulatory subunit [20]. The 20S core subunit can confer the proteolytic activities of 26S proteasome, whereas the 19S regulatory subunit plays a crucial role in the ubiquitin protein conjugates via ATP-dependence pathway [7, 21–22]. Along with the extensive research towards the 26S proteasome, it has been believed to affect an ATP-dependent proteolytic degradation of many proteins including cyclin-dependent kinase inhibitors, transcription factors, cell cycle specific cyclins, ornithine decarboxylase, oncoproteins and other pivotal regulatory cellular events [7]. Due to the direct or indirect regulatory impact on these cellular proteins related to the cancer progression, more attention has been paid to the proteasome for the targeted cancer therapy [23–29]. Based on this anti-cancer target, some of the proteasome inhibitors have been performed and tried to influence on the active sites of 26S proteasome of tumors [25–28]. It was said that proteasome inhibitors were able to increase apoptosis in different tumors [29]. Moreover, inhibiting proteasome could result in cell cycle arrest, decreasing cell adhesion, suppression of cell migration, reducing angiogenesis, interrupting DNA repair response and damaging immune and inflammatory responses [7]. Now, more and more proteasome inhibitors are selected to treat malignancy diseases in clinical trials [30–31]. New type proteasome inhibitors with various efficiencies are expected to be developed and tested for activities of anti-tumors.

Regarding current knowledge, PSMC2, encoding an essential regulatory complex of the 19S proteasome, determines the quantity of the 26S proteasome and regulates catalyzing the unfolding and translocation of subunits into the 20S proteasome [11]. Based on the intimate relationship between PSMC2 and the 26S proteasome, oncology researchers launched a span-new theoretical branch including PSMC2 and malignancy. Nijhawan first disclosed that the therapeutic efficacy of PSMC2 suppression for ovarian cancer *in vivo* [11]. Nevertheless, more extensive and systematic researches on the functional roles of PSMC2 in osteosarcoma are still lacking. Therefore, we carried out an elaborate research to explore the correlation of PSMC2 with human osteosarcoma.

This study verified the hypothesis that PSMC2 was high-expressed in human osteosarcoma and promoted tumorigenesis. Using tissue microarray experiments, we showed that PSMC2 protein was detected in 16 (66.7%) osteosarcoma specimens and mainly showed positive expression in nucleus and cytoplasm. More importantly, this study indicated that abnormal expression of PSMC2 might be related to aggressive tumor features, including histological types and TNM stages. To demonstrate the expression levels of PSMC2 in different osteosarcoma cell lines, we used real time-PCR and western blotting to verify that PSMC2 was ubiquitously expressed in SaoS-2, MG-63, U-2OS and HOS osteosarcoma cell lines. These results suggested that PSMC2 might be involved in osteosarcoma pathogenesis.

Furthermore, we found the necessary function of PSMC2 in osteosarcoma cell proliferation, apoptosis, colony forming, migration, invasion and tumorigenesis via loss-of-functional studies. Tumor cells usually possess the capacities of infinite proliferation, rare apoptosis and rapid colony forming. Compared with normal osteosarcoma cells, the growth of osteosarcoma cells was significantly reduced after PSMC2-knockdown. Moreover, silencing of PSMC2 induced osteosarcoma cells arrest in G_2_/M and S phases as well as significantly increased apoptosis. Additionally, PSMC2-knockdown cells were less prone to form single cell derived colonies. As the other two important properties of the malignancy, tumorigenesis and metastasis were separately tested by *in vivo* and *in vitro* experiments. Osteosarcoma xenograft mouse models strongly supported the critical role of PSMC2 expression for in vivo tumorigenesis in nude mice. Using tests of wound-healing assay and transwell migration, a positive correlation between PSMC2 expression and osteosarcoma metastasis had also been demonstrated. Taken together, PSMC2 could potently facilitate tumorigenesis and invasion in many respects throughout the progression of osteosarcoma.

To exclude the impact of blocking proteasome and overexpressing PSMC2, we did a related functional experiment, respectively. Proteasome is irreplaceable in cells and consist of PSMC2 and other ingredients. In this study, inhibiting proteasome resulted in cell cycle arrest, cells apoptosis and decreased colony forming, verifying the importance of proteasome for osteosarcoma cell biological behaviors. To the certain extent, suppressing PSMC2 expression showed the partial or whole effect on inhibiting proteasome activity. Moreover, the result of overexpressing PSMC2 in osteosarcoma cells is still secret. We established the overexpressing PSMC2 cells using lentivirus transfection. For this section, the results also presented inhibitive effect of proliferation and colony forming. Unexpectedly, exogenously increased expression of PSMC2 would also inhibit proliferation and colony formation in osteosarcoma cells. We noticed Nijhawan et al. has reported that partial genomic loss of PSMC2 was discovered in more than 3000 tumors [11]. Herein, we may suggest that overexpressing PSMC2 may destroy malignant features and only partial expression of PSMC2 is an actual situation in the osteosarcoma cells. Interestingly, this partial expression of PSMC2 in osteosarcoma is still stronger than the expression of PSMC2 in normal tissue according to the results of our tissue microarray. The correlation of the PSMC2 expression and malignance degree of osteosarcoma needs further study.

To investigate the molecular tumorigenesis mechanisms of PSMC2; we have used whole-genome Affymetrix GeneChip analysis to explore the striking changes of cancer-related genes between normal osteosarcoma cells and PSMC2 knockdown cells. ITGA6, FN1, CCND1, CCNE2 and TGFβR2 from KEGG_PATHWAYS_IN_CANCER were subsequently picked up by the gene set enrichment analysis (GO and KEGG pathway analysis). At the transcription level, the mRNAs of five genes were markedly down-regulated by PSMC2 silencing. Consequently, the reduction in proteins expression of those genes was further confirmed by western blotting. ITGA6 has been found to associate with tumor cell adhesion, metastasis and resistance to the drugs 32-33, leading to poor prognosis [33]. We determined a positive correlation between PSMC2 and ITGA6. For the malignancies, FN1 is regarded as critical down-regulated genes involving in cell migration and invasion [34–35]. In our study, silencing PSMC2 leads to the decrease in FN1 expression, which might contribute to PSMC2 depletion attenuated cell migration. It is quilt accepted that alterations in the CCND1 and CCNE2 gene affect the cell cycle and are frequently observed in various malignancies [36–39]. CCND1 and CCNE2 have been verified to essentially direct cell cycle through the late G1 and early S phase. Here, we found that the expression of CCND1 and CCNE2 was decreased by silencing PSMC2. Unexpectedly, G2/M phase arrest in PSMC2-siRNA Sao-S cells and G2/M and S phases arrest in PSMC2-siRNA MG-63 cells were observed. In fact, this phenomenon is not very rare. González-Sarrías et al. found significant G2/M arrest in MCF7 breast cancer cells with decreasing the CCND1 protein level by applying maple polyphenols, ginnalins A-C [40]. KHF16, the main structure of Cimicifuga foetida could the downregulated the CCND1 expression and caused the G2/M arrest in multiple ERα/PR/HER2 triple-negative breast cancer cell lines [41]. Unfortunately, this phenomenon involving CCNE2 in other articles has not been reported. We thought that silencing PSMC2 might lead to an extensive and intricate chaos of cell cycle in osteosarcoma cells. However, due to the limitation of selected genes by microarrays, this phenomenon could not be explained in this study. TGFβR2, a key mediator of TGFβ signalling, was discovered to increase tumor metastatic and invasive abilities [42]. Here, we presented the silence of PSMC2 would effectively suppress osteosarcoma though TGFβR2 expression was reduced by PSMC2 knockdown. Additionally, by IPA we found a large-scale of genes involved in PSMC2 centralized networks, which were still worthy of being studied in the future.

Overall, this study has indicated some new and key findings, as follows. (I) The expression is low in normal bone tissue while is higher expressed in examined osteosarcoma tissues. (II) PSMC2 plays pivotal roles in osteosarcoma cell proliferation, apoptosis, colony forming, migration, invasion and tumorigenesis; (III) In osteosarcoma cells, the expression of PSMC2 is modest and adequate for osteosarcoma malignant feature; (IV) PSMC2 mainly plays the carcinogenesis role in osteosarcoma by regulating cancer-related genes including ITGA6, FN1, CCND1, CCNE2 and TGFβR2. Meanwhile, it may also participate in other cancer-associated pathways. Therefore, the finding of this study provided the new insight into the mechanism how PSMC2 contribute to osteosarcoma malignancy and further suggested PSMC2 might serve as an attractive potential therapeutic target drug target for osteosarcoma treatment.

## MATERIALS AND METHODS

### Ethics statement

The Ethics Committees of the First Affiliated Hospital of Dalian Medical University have approved all studies about human participants. All participants have written informed consents.

### Patient samples

Twenty-four cases of osteosarcoma tissues and 5 cases of normal bone tissues were obtained from the First Affiliated Hospital of Dalian Medical University and selected for this study. After surgical resection, all the osteosarcoma and normal bone tissues were frozen within 30 minutes.

### PSMC2 immunohistochemistry analysis

Immunohistochemistry staining was used via following the standard methods. In brief, 4-mm-thick paraffin tissue samples were deparaffinized in Histoclear and hydrated through graded alcohol. In a pressure cooker, antigen retrieval was accomplished by heating the slice at 120°C in pH 6 target retrieval solution for 10 minutes. We incubated primary antibodies for one hour in a humidification chamber at room temperature. Then secondary antibody conjugated with horseradish peroxidase was incubated for 30 minutes at room temperature. 3,3-diaminobenzidine was used to indicate antibody binding, and the binding reaction could be terminated by distilled water when brown colour appeared. Counterstain with hematoxylin, dehydration in graded alcohol and mounting were performed in order. Rabbit polyclonal anti-PSMC2 antibody (Biorbyt Ltd, Cambridge, Cambridgeshire, UK) was used. All reagents for immunohistochemistry were from Maixin (Maixin Biotech Co, Fuzhou, Fujian, China). For each run of immunohistochemistry, appropriate positive and negative controls were performed. In immunohistochemistry microarray, the PSMC expression levels were scored based on the percentage of osteosarcoma cells stained positive for PSMC2, with (−) denoting 0.0-5.0% of osteosarcoma cells stained, (+) denoting 5.0-30.0% of osteosarcoma cells stained, (++) denoting 31.0-50.0% of osteosarcoma cells stained and (+++) denoting 51.0-80.0% of osteosarcoma cells stained.

### Cell and cells culture

Osteosarcoma cell lines that included SaoS-2, U-2OS, HOS and MG-63 were obtained from China Center Type Culture Collection (CCTCC, Shanghai, China). Osteosarcoma cell lines were cultured in Dulbecco's modified Eagle's medium, Eagle's minimal essential medium, DMEM-F12 growth medium and McCoy's 5A medium mixed with 10.0% fetal bovine serum (FBS) that were purchased from Gibco (Invitrogen, Carlsbad, CA, USA). Cells were incubated in a 37°C, 5.0% CO_2_ humidified incubator.

### PSMC2 gene knockdown and up-regulation

To silence and increase expression of PSMC2 gene, osteosarcoma cells were transfected with lentivirus. The small interference RNA (siRNA) was designed to target the human PSMC2 gene (Gene ID, 5701). The siRNA sequence targeting PSMC2 is as follow. si-PSMC2: 5-GCCAGGGAGATTGGATAGAAA-3. The lentivirus without siRNA insert was used as a control. The process of siRNA constructs consists of synthesizing and cloning into the pGCSIL-green fluorescent protein (GFP) plasmid vector with Age I/EcoRI sites (GeneChem, Shanghai, China). Lentivirus was packaged into 293 T cells using virus titers and Lipofectamine 2000 (Invitrogen, Carlsbad, CA, USA) as soon as cell density reached 70%. The interference efficiency of PSMC2-siRNA in 293 T cells was tested by GFP. On the third day after transfection, we collected the lentivirus particles expressing PSMC2-siRNA from cell culture medium. The centrifugal ultrafiltration device (Millipore, Billerica, MA, USA) was used to concentrate lentivirus, which was then preserved at −80°C. Then SaoS-2 cells and MG-63 cells were cultured in 6-well plates until grew to 30% confluence. Then, they were infected with a suitable quantity of recombinant lentivirus containing PSMC2-siRNA or nontargeting siRNA at 37°C in the presence of 6 μg/ml polybrene (Sigma-Aldrich, St Louis, MO, USA), respectively. Construction of recombinant lentivirus (LV-PSMC2 (19674-1)) containing PSMC2 (GeneChem, Shanghai, China) was also accomplished by the similar method above. After lentivirus construction, SaoS-2 cells were selected to be transfected with lentivirus for establishing the over-expressing PSMC2 osteosarcoma cells.

During all the process of this study, cells transfected with empty GFP lentivirus were selected as the negative control group.

### Inhibition of proteasome in osteosarcoma cells

The most common agent to inhibit proteasome in experiments is MG132 (a selective inhibitor of proteasome, Sigma-Aldrich, St Louis, MO, USA). We selected 30μm/L MG132 as the work concentration thought MTT assay. Accordingly, SaoS-2 osteosarcoma cells treated with MG132 were regarded as proteasome-blocking cells.

### Proliferation was determined by Cellomics ArrayScan and MTT assay

A high content cell imaging algorithm of Cellomics ArrayScan (Thermo Fisher Scientific Inc., DE, USA) was used to count cells once per day from the second day when the cells were seeded in the 96-well culture plate. Finally, the growth curve was made to judge the cell proliferation. Moreover, MTT assay was selected to cells applied with different intervention for checking proliferation through comparing optical density.

### Xenotransplant murine models

We carried out animal xenograft model studies by institutional guidelines; The right armpit region of 6-week female nude mice was given the subcutaneous injection with SaoS-2 cells (2 × 10^5^). The injection frequency was every three days, and the total of injection was six times. On the 11th day after injection, we measured tumor diameters every two days by NightOWL LB983 (Berthold Technologies, Germany). Mice were killed on the 35th day after injection. Then the tumors were obtained and weighted following necropsy. Finally, the formula (length × width2 × ½ mm^3^) was used to calculate tumor volume.

### Western blotting

Phosphate-buffered saline was selected to wash cells. Whole cell lysates of osteosarcoma cells were processed in lysis buffer (1.0% Nonidet P-40, 50mM Tris-HCl (pH 8.0), 100mM sodium fluoride, pyrophosphate, 30mM sodium, 5mM EDTA, 2mM sodium orthovanadate and 2mM sodium molybdate) containing protease inhibitors (1mM phenylmethylsulfonyl fluoride, 10 mg/ml leupeptin and 10 mg/ml aprotinin). SDS–PAGE loading buffer was used to scrape the lysates that were then boiled for 5 minutes. Protein Assay (Bio-Rad, Hercules, CA, USA) was selected to determine protein concentrations. We loaded 20 mg protein on the 8.0-12.0% Bis-Tris gel (NuPAGE; Invitrogen, Carlsbad, CA, USA) and then blotted the protein onto a nylon membrane. Ponceau S (Sigma-Aldrich, St Louis, MO, USA) staining the nylon membranes could indicated the adequate protein transfer. Then, the primary antibodies below were chosen: antibody raised against PSMC2, CCND1, CCNE2, ITGA6, TGFβR2 (rabbit polyclonal, Abcam plc, Cambridge, UK) and FN1 (mouse polyclonal, Abcam plc, Cambridge, UK). The second antibodies included Glyceraldehyde 3-phosphate dehydrogenase (GAPDH) (rabbit monoclonal, Santa Cruz Biotechnology, Santa Cruz, CA, USA) and β-actin (mouse monoclonal, Santa Cruz Biotechnology, Santa Cruz, CA, USA). Enhanced chemiluminescence (Amersham Pharmacia Biotechnology, Piscataway, NJ, USA) and Fuji LAS1000 Plus chemiluminescence imaging system (Kodak, Stamford, CT, USA) was used to perform the detection.

### RNA isolation and real-time PCR

Trizol Reagents (Invitrogen, Carlsbad, CA, USA) was selected for isolating total RNA from cells of different groups. And templates of cDNA were accomplished via real-time PCR using cDNA M-MLV kit (Promega, Fitchburg, WI, USA). Then the products were used to generate real-time PCR templates. Primer pairs for real-time PCR with SYBR Master Mixture (Takara Biotech, Dalian, China) monitored gene expression. Real-time PCR machine TP800 (Takara Biotech) was responsible for analyzing the results. PCR conditions were strictly controlled as below: ten minutes at 95°C followed by 40 cycles of 15 seconds at 95°C and 60 seconds at 60°C. Compared with control, the fold changes of mRNA levels were calculated via the Δ ΔCt method. GAPDH was used as an internal reference for normalization.

### Cell cycle analysis and apoptosis measurements

Ice-cold 70.0% alcohol was used to fix and permeabilize cells at 4°C overnight. Washed with phosphate buffer, cells were dealt with RNase for 20 minutes at 37°C and then stained with 40μg/ml propidium iodide. FACSCalibur flow cytometer (BD, Franklin Lakes, NJ, USA) was used to analyze DNA content of cells. For each sample, a total of 10000 events were counted. The percentage of cells with different phase (G1, S and G2/M) was analyzed and determine by CellQuest software (BD, Franklin Lakes, NJ, USA). Apoptosis was assessed with Annexin VAPC Apoptosis Detection Kit 88-8007 (eBioscience Inc, San Diego, CA, USA) and flow cytometric analysis.

### Colony formation assay

We used a soft agar assay to assess the function of PSMC2 on colony forming of osteosarcoma cells. Incubated for 15 days, Giemsa (Sigma-Aldrich, St Louis, MO, USA) was performed to stain colonies for 30 minutes after cells fixation with 4.0% paraformaldehyde for 15 minutes. Colonies that were bigger than 100 μm diameter wound be counted.

### Microarrays and data analysis

Trizol Reagents (Invitrogen, Carlsbad, CA, USA) was used for isolating total RNA from cells of different groups. Then NanoDrop 2000 (Thermo Fisher Scientific Inc., DE, USA) and Agilent Bioanalyzer 2100 (Agilent Technologies Inc., Santa Clara, CA, USA) were performed to assess RNA integrity. GeneChip® 3′IVT Express Kit (Affymetrix Inc., Santa Clara, CA, USA) was chosen to amplify extracted RNA samples, which were then hybridised onto Affymetrix GeneChip® 133 Plus 2.0 Arrays (Affymetrix Inc., Santa Clara, CA, USA). We used standard Affymetrix protocols to hybridize and scan the arrays. Affymetrix GeneChip Scanner 3000 and Affymetrix GeneChip Command ConsoleTM 1.1 software were performed to scan and analyze the image signal, respectively. Finally, the image signal was transformed into digital information and analyzed via SAM software.

We performed GO enrichment analysis to explore biological functions of the PSMC2 in osteosarcoma cell. GO, as a common gene analysis method, can provide functional annotation and descriptive framework of the gene sets data. We also used KEGG and Biocarta pathways enrichment analysis to find important pathways associated with for PSMC2 in osteosarcoma. Biocarta (http://www.biocarta.com/) and KEGG (www.genome.jp/kegg/pathway.html) pathways databases are recognized and comprehensive databases including all kinds of biochemistry pathways. R software, version 3.0.1 was utilized in this analysis. Additionally, for tentative exploration of the proteins networks, IPA had been done. IPA Software was purchased from Ingenuity Systems (Redwood City, CA, USA).

### Cell wound-healing assay

Osteosarcoma cells were incubated in 6-well plates until 80% confluence. A 10-μL tip was used to generate scratch-wound. After 12 hours, the wounded cells were recorded by taking photographs. By measuring gap sizes, cell migration was evaluated in multiple fields.

### Transwell invasion assay

Transwell membranes precoated with Matrigel (BD Biosciences, Bedford, MA, USA) were used to assess cell invasion. Cells of different groups were plated separately at a density of 5 × 10^4^ cells for each well. 30.0% FBS was then added to the lower chamber. After 24 hours incubation, cells remaining in the upper chamber were removed, while invading cells were fixed with 3.0% paraformaldehyde, stained with Giemsa stain (Sigma-Aldrich, St Louis, MO, USA).

### Statistical analysis

All of the data were presented as Means ± SD. Three repeated experiments for each group were done. Student's t-test (SPSS 17.0 software), Fisher's exact test (SPSS 17.0 software) and one-way analysis of variance (SPSS 17.0 software) were used to analyze and determine the statistical significance. P<0.05 was considered as significant.
